# Magnitude and factors associated with anemia among pregnant women attending antenatal care in Bench Maji, Keffa and Sheka zones of public hospitals, Southwest, Ethiopia, 2018: A cross -sectional study

**DOI:** 10.1371/journal.pone.0225148

**Published:** 2019-11-21

**Authors:** Tesfaye Abera Gudeta, Tilahun Mekonnen Regassa, Alemayehu Sayih Belay

**Affiliations:** 1 Department of Nursing (Maternal Health Unit), College of Health Science, Mizan-Tepi University, Mizan-Aman, Ethiopia; 2 Department of Nursing (Adult Health Unit), College of Health Science, Mizan-Tepi University, Mizan-Aman, Ethiopia; Anglia Ruskin University, UNITED KINGDOM

## Abstract

**Background:**

Anemia during pregnancy is a common public health problem globally and it defined as the hemoglobin concentration of less than 11 g/dl. Anemia during pregnancy has maternal and perinatal diverse consequences and it increase the risk of maternal and perinatal mortality. The aim of this study is to assess magnitude and factors associated with anemia among pregnant women attending antenatal care in Bench Maji, Keffa and Sheka zones of public hospitals, South west, Ethiopia, 2018.

**Methods:**

A cross-sectional study was employed on 1871 pregnant mothers from selected hospitals. All third trimester pregnant women attending antenatal care at Mizan-Tepi University Teaching Hospital, Tepi, Gebretsadik Shawo and Wacha public hospitals were included in the study. Data was entered to Epidata version 3.1 and exported to SPSS version 21 for analysis. Logistic regression analysis was carried out to identify independently associate factors at confidence interval of 95% and significance level of P-value <0.05.

**Result:**

The magnitude of anemia in this study from the total study participant was 356 (19.0%). Among anemic pregnant women, 330 (92.7%), 21(5.9%) and 5(1.4%) were mild anemia, moderate anemia and severe anemia respectively. Age group 20–24 [AOR 6.28(2.40–16.42)], 25–29 [AOR = 6.38 (2.71–15.01)], 30–34 [AOR = 5.13 (2.27–11.58) and age ≥35 years [AOR = 2.53 (1.07–5.98)], educational status (read and write) [AOR 2.06, 95% CI (1.12–3.80)], gestational age(term)[AOR 1.94, 95% CI (1.27–2.96)], Caffeine (coffee and tea) and alcohol use occasionally [AOR 2.01, 95% CI (1.14–3.55)] and [AOR 2.59, 95% CI (1.49–4.52)] respectively, nutritional status (under nutrition) [AOR 3.00, 95% CI (2.22–3.97)] and family size (>6) [AOR 2.66, 95% CI (1.49–4.77)] were factors associated with anemia.

**Conclusion:**

The magnitude of anemia found to be high. Age, educational status of the mother, gestational age, caffeine and alcohol use, Nutritional status and family size were factors significantly associated with anemia. To prevent adverse outcome of anemia, health care providers should work on these factors.

## Introduction

World Health Organization (WHO) has defined anemia during pregnancy as the hemoglobin concentration of less than 11 g/dl [1]. Depending on hemoglobin concentration, anemia during pregnancy is classified as severe if the hemoglobin level is less than 7.0 g/dl, moderate when it falls between 7.0–9.9 g/dl, and mild from 10.0–11 g/dl [2–4]. The symptoms and signs of anemia are vague and nonspecific, including pallor, easy fatigability, headache, palpitations, tachycardia, and dyspnea. Angular stomatitis, glossitis, and koilonychia (spoon nails) may be present in long-standing severe anemia [5].

According to WHO, anemia is considered of a severe public health implication if its rate of ≥40% [6]. Anemia during pregnancy is a main public health problem worldwide, particularly in developing countries where there is inadequate diet and poor prenatal vitamins and iron and folic acid intake[7] and it affects the physical health and mental development of individual causing low productivity and poor economic development of a country[7,8].

Globally, every year anemia causes more than 115,000 maternal and 591,000 perinatal deaths [3]. Worldwide, anemia affects more than half billion reproductive age women [9–12]. It is the most common problem during pregnancy, therefore, 56% of pregnant women in low and middle income countries have anemia. Because of this reason, anemia during pregnancy contributes to 23% of indirect causes of maternal deaths in developing countries [8].

The prevalence of anemia was found be highest among pregnant women in developing countries, particularly in sub- Sahara Africa (57%), in South-East Asia (48%) and lowest prevalence (24.1%) was reported among pregnant women in South America [6].

Anemia in pregnancy has maternal and perinatal diverse consequences and it increase the risk of maternal and perinatal mortality [13, 14]. It also brings different obstetrical problems like; prematurity, low birth weight[15], abortion, intrauterine fetal death and perinatal mortality [16] and other maternal health problems like; impaired immune function, poor work capacity, fatigue, increased risk of cardiac diseases and mortality[8,14].

Even though there is different contributing factors for anemia like genetic, nutritional, and infectious disease factors, iron deficiency is the most common cause of 75% of anemia cases [8,17–20]. Iron deficiency anemia is common in pregnant women and it affects the development of the once country through decreasing the physical and cognitive development of children and productivity of adults [20].

The prevalence of anemia in pregnancy has remained unacceptably high and still it is a major public health concern in Ethiopia despite the fact that routine iron and folic acid supplementation during pregnancy was provided by the skilled providers [21]. This is due to the fact that poor nutritional intake, repeated infections, menstrual blood loss, and frequent pregnancies are common in Ethiopia which is associated with poor socio economic conditions during pregnancy [22, 23] and poor antenatal care follows up during pregnancy [24].

In Ethiopia, about 17% of reproductive age women are anemic and 22% of them were pregnant [25]. Despite its known adverse effect on the pregnant women and children, there is no updated data available in the study area. Since no study was conducted in the study area, the finding of this study will be important to design appropriate interventions to reduce the high burden of the disease in the area and country at large. Therefore, this study is aimed at determining the magnitude of anemia in pregnant women and identifying its associated factors in the hospitals of Bench Maji, Keffa and Sheka zones Southwest Ethiopia.

## Methods and materials

### Study area and period

The study was conducted in public hospitals of Bench Maji, Sheka and Keffa zones namely, Mizan Tepi University teaching hospital (MTUTH), Tepi general hospital, Wacha hospital and Gebretsadik Shawo hospital from January 15- March 30/2018. MTUTH is located in Bench Maji zone on 560 kms from Addis Ababa. The two hospitals: Gebretsadik Shawo and Wacha hospitals are found in kefa zone at a distance of 441 and 520 kms away from Addis Ababa respectively, while Tepi general hospital is located in Sheka zone, 565 Kms away from capital city of Ethiopia, Addis Ababa.

### Study design

Facility based cross-sectional study design was used.

### Source and study population

All pregnant women who attending antenatal care at MTUTH, Tepi hospital, Gebretsadik Shawo hospital and Wacha hospital were considered as source of population and all pregnant women those fulfilled the inclusion criteria were considered as study population.

### Inclusion and exclusion criteria

All third trimester pregnant women attending antenatal care at MTUTH, Tepi, Gebretsadik Shawo and Wacha public hospitals were included in the study; however pregnant women who were critically ill and unable to communicate during data collection were excluded from the study.

### Sample size determination

The sample size was determined by using a single population proportion sample size calculation formula considering the following assumptions. d = margin of error of 2% with 95% confidence interval, estimated prevalence of anaemia is 23% [26] and considering non response rate of 10%. Then the final sample size became 1871.

### Sampling technique

All hospitals found in three zones were included in the study. The total sample size (1871) was allocated to the four public hospitals. The sample size allocation was based on the source of population from each hospital. The source of population of each hospital was taken from antenatal follow up report. Then the average was considered as source of population. The study participants were consecutively taken from each hospital until the sample size was achieved.

### Operational definitions and definition of terms

**Anemia in pregnancy**: In this study, anemia defined as hemoglobin level less 11 g/dl during third trimester. Woman with hemoglobin less 11g/dl was coded 1 whereas woman who was not anemic coded as 0.

**Pregnant women** are classified as non-anemic if hemoglobin ≥11.0 g/dl, mild anemic if the range is 10 to 10.9 g/dl, moderate anemic if the range is 7 to 9.9 g/dl and severely anemic if hemoglobin is below 7.0 g/dl.

### Data collection instruments/tool

The data was collected using pre-tested questionnaire and anthropometric measurements. The questionnaire was developed based on tools that were applied in different related literatures (12–18). Questionnaires were developed in English and translated to Amharic by experts and translated back to English to see consistency of the question. The questionnaire contains sections for assessing anemia, demographics and associated factors.

### Data collectors

Twelve data collectors who bachelor degree holder midwives were recruited. Four supervisors who had master degree holders in maternal health were recruited.

### Data collection procedure

Data was collected through face to face interview, measurements and reviewing of medical record of the mother by using pre-tested structured questionnaire and check list by trained data collectors. Last normal menstrual period (LNMP) was confirmed from her chart and client report. Gestational age was calculated based on the last normal menstrual period (LNMP).

When LNMP-based gestational age is unknown, we relied on obstetric ultrasonography measures. Nutritional status was assessed by using Mid-upper arm circumference (MUAC) measurement. MUAC < 21cm considered as undernourished.

### Data processing and analysis

EPI data Statistical software version 3.1 and Statistical Package for Social Sciences (SPSS) software version 21.0 was used for data entry and analysis. After organizing and cleaning the data, frequencies & percentages was calculated to all variables that are related to the objectives of the study. Variables with P- value of less than 0.25 in binary logistic regression analysis was entered into the multivariable logistic regression analysis to control confounds so that the separate effects of the various factors associated with anemia could be assessed. Odds ratio with 95% confidence interval was used to examine associations between dependent & independent variables. P value less than 0.05 was considered significant. Finally the result was presented by using tables, charts and narrative form.

### Data quality control measures

The quality of the data was assured by using validated pre-tested questionnaires. Prior to the actual data collection, pre-test was done on 5% of the total study eligible subjects and have similar characteristics at Mizan health center and necessary amendments was made.

The validity of the tool was checked by face validity. Data collectors were trained intensively on the study instrument and data collection procedure that includes the relevance of the study, objective of the study, confidentiality, informed consent and interview technique. The data collectors worked under close supervision of the supervisors to ensure adherence to correct data collection procedures.

Supervisors checked the filled questionnaires daily for completeness. Every morning, supervisors and data collectors conducted morning session to solve if there is any faced problem as early as possible and to take corrective measures accordingly. Moreover, the data was carefully entered and cleaned before the beginning of the analysis.

### Ethical considerations

Ethical approval was obtained from Mizan-Tepi University. Further permission was obtained from each hospital. After explaining the objectives of the study in detail, written informed consent was taken from all study participants.

## Result

### Socio-demographic characteristics

All the sampled mothers were participated (100% response rate). A total of 853(45.6%) participants were rural residents, 481(25.7%) were illiterates, 1808(96.7%) were married, 483(79.3%) were house wives and the family size of 1421(75.9%) participants were four children or less (Table 1).

**Table 1:** Socio-demographic characteristics of women attending antenatal care in public hospitals of Benchi-Maji, Kaffa and Sheka zones, Southwest Ethiopia, 2018.

Variables	Category	Frequency	Percent (%)
Age	15–19	168	9.0
	20–24	808	43.2
	25–29	547	29.2
	30–34	221	11.8
	35+	127	6.8
Residence	Rural	853	45.6
	Urban	1018	54.4
Educational status	Unable to read and write	481	25.7
	Able to read write	393	21.0
	Primary education	609	32.5
	Secondary education	246	13.1
	College and above	142	7.6
Marital status	Married	1808	96.7
	Single	36	1.9
	Divorced	5	0.3
	Widowed	10	0.5
	Separate	12	0.6
Religion	Orthodox	837	44.7
	Muslim	387	20.7
	Protestant	637	34.0
	Other	10	0.5
Occupation	Housewife	1483	79.3
	Merchant	170	9.1
	Gov’t employee	117	6.3
	Non-gov’t employee	18	1.0
	Daily labor	83	4.4
Family size	≤4	1421	75.9
	5–6	347	18.5
	≥7	103	5.5

### Variables related to obstetric characteristics

Around half 834 (44.6%) of the study participants were primigravida and almost all 1785(95.4%) of the pregnancy were intended and also 1700 (90.9%) of the pregnancies were term pregnancy.

Majority 1726 (92.3%) of the participants have antenatal care (ANC) follow-up and only 424(25.1%) of participants were started antenatal follow up during their first trimester. And also the majority 1570 (83.9%) were take iron foliate during current pregnancy (Table 2).

**Table 2:** Variables related to obstetric characteristics among women attending ANC in public hospitals of Benchi-Maji, Kaffa and Sheka zones, Southwest, Ethiopia, 2018.

Variables	Category	Frequency	Percent (%)
Gravida	1	834	44.6
	2–4	944	50.5
	>4	93	5.0
Parity	Primiparous	883	47.2
	Multiparous	988	52.8
Pregnancy status	Intended	1785	95.4
	Unintended	86	4.6
Gestational age	Less than 37 weeks	171	9.1
	≥ 37 weeks	1700	90.9
ANC follow-up	Yes	1726	92.3
	No	145	7.7
Among mothers who have ANC follow up, At what month ANC started?	1–3 months	424	25.1
	4–6 months	1185	70.0
	7–9 months	83	4.9
Number of ANC visit	One visit	86	5.0
	Two visit	168	9.7
	Three	423	24.5
	Four and above visit	1049	60.8
Iron foliate intake	Yes	1570	83.9
	No	301	16.1

### Variables related to pregnancy complication and medical illness

A total of 252(13.5%) of participants developed pregnancy-related complication during current pregnancy, 78 (31%), 31(12.3%),21 (8.3%), and 52 (20.6%) were developed preeclampsia, placenta previa, abruptio placenta and antepartum hemorrhage respectively. A total of 281 (15%) faced medical illness during current pregnancy. 1357(72.5%) women were not malnourished based on their mid-upper arm circumference (MUAC) measurement **(**Table 3**).**

**Table 3:** Variables related to pregnancy complication and medical illness among women attending ANC in public hospitals of Benchi-Maji, Kaffa and Sheka zones, Southwest, Ethiopia, 2018.

Variables	Category		Frequency	Percent (%)
Complications on current pregnancy	Yes		252	13.5
	No		1619	86.5
Pregnancy related complications	Gestational hypertension	Yes	5	2
		No	247	98.0
	Preeclampsia	Yes	78	31
		No	174	69.0
	Eclampsia	yes	36	14.3
		No	216	85.7
	Placenta Previa	Yes	31	12.3
		No	221	87.7
	Abruptio placenta	Yes	21	8.3
		No	231	91.7
	Antepartum hemorrhage	Yes	52	20.6
		No	200	79.4
Medical related illness on current pregnancy	Yes		281	15.0
	No		1590	85.0
Medical illnesses	Malaria	Yes	156	8.3
		No	1715	91.7
	HIV	Positive	51	2.7
		Negative	1820	97.3
	ART* status	Started	51	100
Nutritional status (Using MUAC*)	Under nutrition (MUAC<21cm)		514	27.5
	Normal		1357	72.5

*ART **=** Anti-Retroviral Treatment

**MUAC** = Mid-Upper Arm Circumference

### Variables related to behavioral factors

From the total study participants, 1516 (81%) used to drink caffeine (coffee and tea) on a daily basis, and 1272 (68.0%) were never drinking alcohol. Regarding the nutritional status 1252 (66.9%) and 1210 (64.7%) were get dietary counseling and additional diet during current pregnancy respectively (Table 4).

**Table 4:** Variables related to behavioral factors among women attending ANC in public hospitals of Benchi-Maji, Kaffa and Sheka zones, Southwest, Ethiopia, 2018.

Variables	Category	Frequency	Percent (%)
Caffeine intake (coffee & tea) during index pregnancy	Never	167	8.9
	Daily	1516	81.0
	Weekly	28	1.5
	Occasionally	160	8.6
Alcohol intake during index pregnancy	Never	1272	68.0
	Daily	27	1.4
	Weekly	86	4.6
	Occasionally	486	26.0
Mother counseled on dietary practice during current pregnancy	Yes	1252	66.9
	No	619	33.1
Get additional diet during current pregnancy	Yes	1210	64.7
	No	661	35.3
Mothers faced physical harassment during current pregnancy	Yes	129	6.9
	No	1742	93.1

### Magnitude of anemia

**Fig 1:**
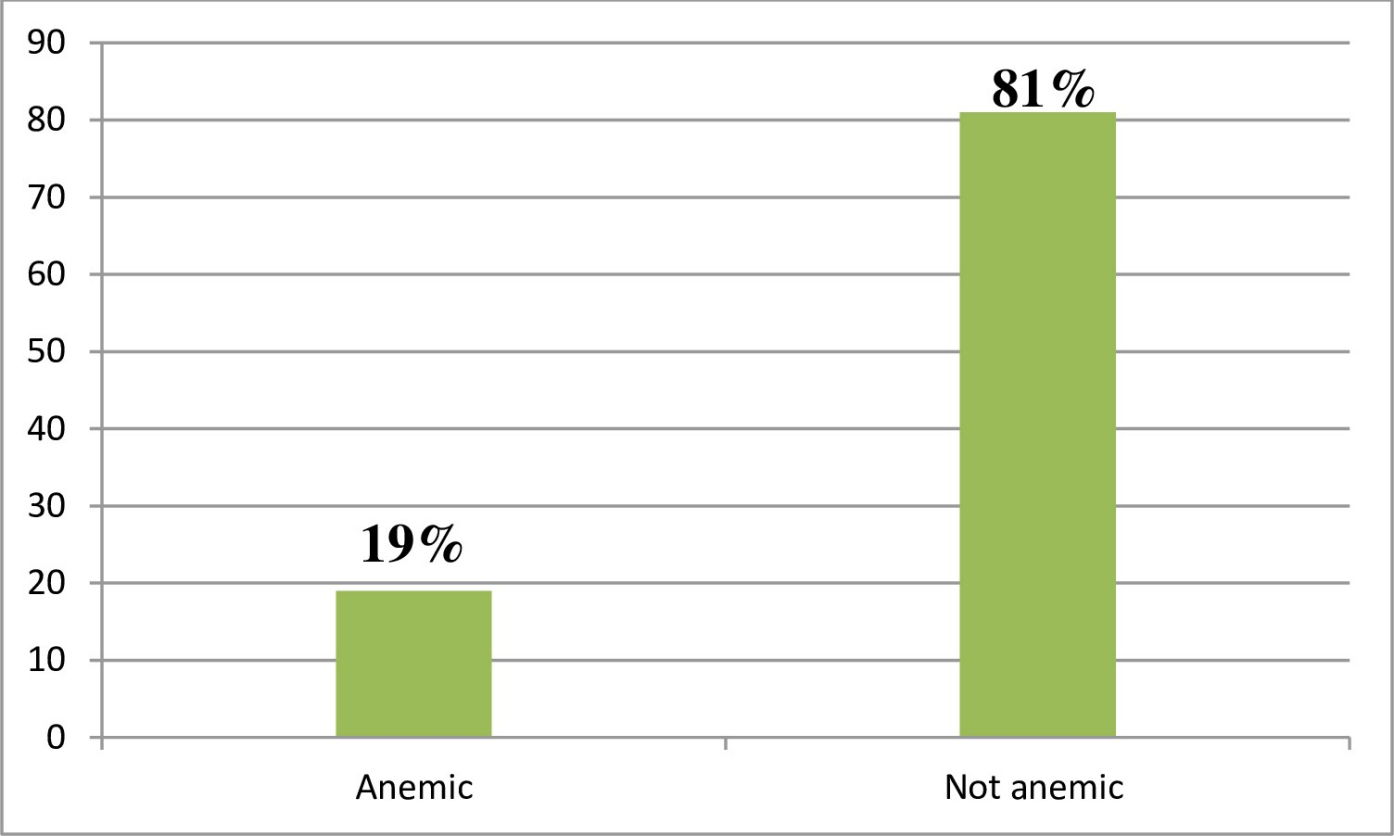
Magnitude of anemia among women attending antenatal care in Bench Maji, Keffa and Sheka zone of public hospitals, Southwest Ethiopia, 2018. This figure shows that the magnitude of anemia in pregnancy which was 19.0%.

The magnitude of anemia in this study from the total study participants (1871) was about 356 (19.0%) at **95 CI** (17.2%-20.7%). Among anemic pregnant women, 330 (92.7%), 21(5.9%) and 5(1.4%) were mild anemia, moderate anemia and severe anemia respectively (Fig 1).

### Factors associated with anemia

Mothers who in age group 20–24 [AOR 6.28 (2.40–16.42)], 25–29 [AOR = 6.38 (2.71–15.01)], 30–34 [AOR = 5.13 (2.27–11.58) and ≥35 years [AOR = 2.53 (1.07–5.98)] were more likely developed anemia as compared to younger age group (15–19). Mothers who have no formal education but read and write were two times more likely to have anemia as compared to mothers whose educational level of diploma and above [AOR 2.06, 95% CI (1.12–3.80)]. A pregnant mother who has gestational age ≥37weeks were two times more likely faced anemia as compared to preterm pregnancy [AOR 1.94, 95% CI (1.27–2.96)]. Pregnant mother who occasionally used caffeine (coffee and tea) and alcohol were two [AOR 2.01, 95% CI (1.14–3.55)] and two & half [AOR 2.59, 95% CI (1.49–4.52)] respectively times more likely developed anemia as compared to mothers never used this substance. Under nourished pregnant women were three times more likely developed anemia as compared to mothers who were well nourished in their nutritional status [AOR 3.00, 95% CI (2.22–3.97)]. Mothers who have larger family size (>6) were three times more likely faced anemia as compared to small family size [AOR 2.66, 95% CI (1.49–4.77)] **(**Table 5**).**

**Table 5:** Factors associated with anemia among mothers attending antenatal care in public hospitals of Benchi-Maji, Kaffa and Sheka zones, Southwest, Ethiopia, 2018.

Variable	Category	Anemia		COR (95% CI)	AOR (95% CI)
		No	Yes		
Age	15–19	132	36	**1**	**1**
	20–24	649	159	0.90(0.60–1.35) 0 0) 0	6.28 (2.40–16.42) *
	25–29	430	117	0.99(0.66–1.52)	6.38 (2.71–15.01)*
	30–34	188	33	0.64(0.38–1.09)	5.13 (2.27–11.58)*
	35+	116	11	0.35(0.17–0.71)	2.53 (1.07–5.98)*
Residence	Rural	647	206	0.54(0.43–0.69)	1.37(0.98–1.92)
	Urban	868	150	**1**	**1**
Educational status	Cannot read and write	355	126	2.30 (1.36–3.88)	1.73(0.923–3.23)
	Read and write	301	92	1.98 (1.16–3.38)	2.06(1.12–3.80)*
	Primary education	535	74	0.90(0.52–1.54)	0.87(0.48–1.62)
	Secondary school	201	45	1.45(0.81–2.59)	1.70(0.89–3.26)
	Diploma and above	123	19	1	1
Parity	Primiparous	740	143	1	1
	Multiparous	775	213	1.42 (1.13–1.80)	1.40 (0.99–1.98)
Gestational age	Preterm (<37weeks)	101	70	1	1
	Term (> = 37 weeks)	1414	286	0.29 (0.21–0.41)	1.94(1.27–2.96)*
ANC follow up	Yes	1432	294	1	**1**
	No	83	62	3.64 (2.56–5.17)	1.56(0.90–2.71)
Intake Iron foliate	Yes	1310	260	1	1
	No	205	96	2.36 (1.79–3.11)	1.26(0.82–1.96)
Current pregnancy complications	Yes	177	75	2.02(1.50–2.72)	1.3610.94–1.98)
	No	1338	281	1	1
Mothers’ HIV status	Negative	1477	343	1	**1**
	Positive	38	13	1.47(0.78–2.80)	1.82(0.89–3.72)
Caffeine intake (Coffee and tea)	Never	136	31	1	**1**
	Daily	1256	260	0.91(0.60–1.37)	0.69 (0.43–1.09)
	Weekly	19	9	2.08(0.86–5.03)	1.61(0.60–4.30)
	Occasionally	104	56	2.36(1.42–3.93)	2.01(1.14–3.55) *
Alcohol intake	Never	1073	199	1	**1**
	Daily	20	7	1.89(0.79–4.52)	1.05(0.45–1.01)
	Weekly	55	31	3.04(1.91–4.84)	1.06 (0.38–3.00)
	Occasionally	367	119	1.75(1.35–2.26)	2.59(1.49–4.52)*
Counseled on dietary practice	Yes	1020	232	1	**1**
	No	495	124	1.10(0.86–1.40)	1.01(0.65–1.56)
Get additional diet during pregnancy	Yes	993	217	1	**1**
	No	522	139	1.22(0.96–1.55)	0.89(0.58–1.35)
Nutritional status	Well-nourished	1171	186	1	1
	Under nourished	344	170	3.11(2.45–3.96)	3.00(2.22–3.97)*
Family size	< = 4	1150	271	1	1
	5–6	291	56	0.82(0.60–1.12)	1.054 .693 1.604
	>6	74	29	1.66(1.06–2.61)	2.66(1.49–4.77)*
History of medical illness	Yes	236	45	0.78(0.56–1.10)	0.76(0.51–1.12)
	No	1279	311	1	1

* = Statistically significant

AOR = Adjusted Adds Ratio, COR = Crude Odds Ratio.

## Discussion

The world health organization estimates that the highest proportion of individuals affected by anemia are in Africa and also in Ethiopia anemia is a severe problem for both pregnant and non-pregnant women of childbearing age [6]. Therefore, this study was planned to assess the magnitude and associated factors of anemia among pregnant women.

The magnitude of anemia in the study area was 19.00%. The magnitude of anemia in pregnant women in this study area is higher than the study done in Addis Ababa (10.1%) [27]. The difference might be to socioeconomic difference, culture of dietary practice and awareness about anemia during pregnancy. The main cause of anemia in pregnancy is nutritional deficiency. So, giving attention during antenatal care about additional diet and supplementation of iron folate are very crucial in reducing the magnitude of anemia among pregnant mothers.

The magnitude of anemia in this study is lower as compared with the studies done in Malaysia (33%) [28], Gana (51%) [29], and in Ethiopia: Tigray(36.1%) [30], Nekemte town (52%) [31], Adama (28.1%) [32], Gode town (56.8%)[33], Bisidimo (27.9) [34], Jijiga town (63.8%)[35] and Ilu Abba bora zone (31.5%) [36]. This difference might be due to the study period and the attention given for focused antenatal care and supplementation of iron sulfate throughout the pregnancy.

The magnitude of this study is consistent with the studies done in India (20%) [37], Mekele town (19.7) [38], Mizan Aman general hospital (23.5%) [39] and Limo district (23%) [26].

In this study, factors influencing magnitude of anemia were identified. Advanced maternal age was statistically associated with anemia during pregnancy. The finding of this study is congruent with the studies done in Ghana and Jijiga [29, 35]. As maternal age increases, the mother may face pregnancy and labour related complications, and other illness which may predispose the mother for anemia. Mothers who haven’t any formal education were more likely develop anemia as compared to formally educated mothers. This finding is similar with study carried out in Malaysia and Tigray [28, 30]. It is obvious that as educational status increases, the life style, socio-economic status and diseases prevention knowledge and skilled also improved.

This study also identified other factors associated with anemia; gestational ages greater or equal to 37weeks were more likely faced anemia. Around 37 and above weeks, the demand of iron is increased which might be the cause for anemia. Mothers who have family size greater than six were more likely develop anemia as compared to mother who have less than five. This finding is consistent with the studies done in Jigjiga and Ilu Abba Bora zone [35, 36].

Pregnant women of Mid Upper Arm Circumference (MUAC) less than 21cm were more likely to be anemic as compared to women not malnourished. The result of this study is comparable with the study conducted in Adama town, Jigjiga town, Gode town and Ilu Abba Bora zone [32–36]. The similarity could be due the facts that under nutrition occur as a result of micro and macro nutrient deficiency and also anemia may occur as complication of malnutrition.

### Strength and limitation of this study

The study was carried out through close supervision and follow up during data collection period, and the analysis was generated from huge sample which increases its representativeness were considered as strength of the study. The study was facility based study; it is difficult to generalize for the community and the study might encounter inter observer error during measurements were considered as limitation of this study.

### Conclusion

The magnitude of anemia found to be high. Age, educational status of the mother, gestational age, caffeine and alcohol use, Nutritional status and family size were factors significantly associated with anemia. To prevent adverse outcome of anemia, health care providers should work on these factors.

## Supporting information

S1 Table Description of variables and measurement for the study in Bench Maji, Keffa and Sheka zones of public hospitals, Southwest, Ethiopia, 2018: This table shows that the description and measurements of dependent and some independent variables. (DOCX)

S1 File Anemia SPSS data. This SPSS data is a data which all statistical analysis was done from it.
(SAV)

## Abbreviations

AOR: Adjusted Odds Ratio
ANC: Ante Natal Care
COR: Crude Odds Ratio
LNMP: Last Normal Menstrual Period
MUAC: Mid Upper Arm Circumference
SPSS: Statistical Package for Social Science
WHO: World Health Organization

## References

[1] Organization WH. The global prevalence of anaemia in 2011. The global prevalence of anaemia in 20112015.

[2] Balarajan Y, Ramakrishnan U, Özaltin E, Shankar AH, Subramanian S. Anaemia in low-income and middle-income countries. The lancet. 2011;378 (9809):2123–35. 10.1016/S0140-6736(10)62304-5 21813172

[3] Salhan S, Tripathi V, Singh R, Gaikwad HS. Evaluation of hematological parameters in partial exchange and packed cell transfusion in treatment of severe anemia in pregnancy. Anemia. 2012;2012. 10.1155/2012/608658 PMC3368167 22693662

[4] Barooti E, Rezazadehkermani M, Sadeghirad B, Motaghipisheh S, Tayeri S, Arabi M, et al Prevalence of iron deficiency anemia among Iranian pregnant women; a systematic review and meta-analysis. Journal of reproduction & infertility. 2010;11(1):17. 23926476 PMC3719272

[5] Paola Aghajanian, Steven W, Mohammed W, Ellene Andrew, Dennis R, et al (2007). Current Diagnosis and Treatment Obstetrics & Gynecology, Tenth Edition, McGraw-Hill Companies.

[6] Benoist Bd, McLean E, Egll I, Cogswell M. Worldwide prevalence of anaemia 1993–2005: WHO global database on anaemia Worldwide prevalence of anaemia 1993–2005: WHO global database on anaemia. 2008.

[7] WHO. The global prevalence of anaemia in 2011. World Health Organization Geneva; 2015.

[8] Black RE, Victora CG, Walker SP, Bhutta ZA, Christian P, De Onis M, et al Maternal and child undernutrition and overweight in low-income and middle-income countries. The lancet. 2013; 382 (9890):427–51. 10.1016/S0140-6736(13)60937-X 23746772

[9] Targets WGN. 2025: Anaemia policy brief. Geneva: World Health Organization 2014.

[10] McClure EM, Goldenberg RL, Dent AE, Meshnick SR. A systematic review of the impact of malaria prevention in pregnancy on low birth weight and maternal anemia. International Journal of Gynecology & Obstetrics. 2013;121(2):103–9. 23490427 10.1016/j.ijgo.2012.12.014

[11] Lee AI, Okam MM. Anemia in pregnancy. Hematology/Oncology Clinics. 2011;25(2):241–59. 10.1016/j.hoc.2011.02.001 21444028

[12] Karaoglu L, Pehlivan E, Egri M, Deprem C, Gunes G, Genc MF, et al The prevalence of nutritional anemia in pregnancy in an east Anatolian province, Turkey. BMC Public Health. 2010;10(1):329 20537176 10.1186/1471-2458-10-329 PMC2904273

[13] Levy A, Fraser D, Katz M, Mazor M, Sheiner E. Maternal anemia during pregnancy is an independent risk factor for low birthweight and preterm delivery. European Journal of Obstetrics & Gynecology and Reproductive Biology. 2005; 122(2):182–6. 16219519 10.1016/j.ejogrb.2005.02.015

[14] Adam I, Ali AA. Anemia during pregnancy Nutritional Deficiency: InTech; 2016.

[15] Peña-Rosas JP, Viteri FE. Effects and safety of preventive oral iron or iron+ folic acid supplementation for women during pregnancy. Cochrane Database Syst Rev. 2009;4: CD004736. 10.1002/14651858.CD004736.pub3 19821332

[16] Sifakis S, Pharmakides G. Anemia in pregnancy. Annals of the New York Academy of Sciences. 2000;900(1):125–36. 10818399 10.1111/j.1749-6632.2000.tb06223.x

[17] Baig-Ansari N, Badruddin SH, Karmaliani R, Harris H, Jehan I, Pasha O, et al Anemia prevalence and risk factors in pregnant women in an urban area of Pakistan. Food and nutrition bulletin. 2008;29(2):132–9. 10.1177/156482650802900207 18693477 PMC3917507

[18] Haidar J. Prevalence of anaemia, deficiencies of iron and folic acid and their determinants in Ethiopian women. Journal of health, population, and nutrition. 2010;28(4):359 10.3329/jhpn.v28i4.6042 20824979 PMC2965327

[19] Akhtar M, Hassan I. Severe anaemia during late pregnancy. Case reports in obstetrics and gynecology. 2012;2012. 10.1155/2012/485452 PMC3439950 22988533

[20] Viveki R, Halappanavar A, Viveki P, Halki S, Maled V, Deshpande P. Prevalence of anaemia and its epidemiological determinants in pregnant women. Al Ameen J Med Sci. 2012;5(3):216–23.

[21] Government of the Federal Democratic Republic of Ethiopia National Nutrition Programme 2008–2015 Available from: https://www.usaid.gov/documents/1867/government-federal-democratic-republic-ethiopia-national-nutrition-programme.

[22] Abriha A, Yesuf ME, Wassie MM. Prevalence and associated factors of anemia among pregnant women of Mekelle town: a cross sectional study. BMC research notes. 2014;7(1):888. 25487251 10.1186/1756-0500-7-888 PMC4295569

[23] Argaw B, Argaw-Denboba A, Taye B, Worku A. Major risk factors predicting anemia development during pregnancy: unmatched-case control study. J Community Med Health Educ. 2015;5(353):2161–0711.1000353.

[24] Central Statistical Agency (CSA) and ICF, Ethiopia Demographic and Health Survey 2016: Key Indicators Report, Central Statistical Agency and and ICF, Addis Ababa, Ethiopia, and Rockville, MD, USA 2016.

[25] CSA I. Ethiopia demographic and health survey 2011. Addis Ababa, Ethiopia and Calverton, Maryland, USA: Central Statistical Agency and ICF International 2012;430.

[26] Lebso M, Anato A, Loha E (2017) Prevalence of anemia and associated factors among pregnant women in Southern Ethiopia: A community based cross-sectional study. PLoS ONE 12(12): e0188783 10.1371/journal.pone.0188783 29228009 PMC5724831

[27] Kitila KT, Tulu BL, Bedaso DG, Negwo DT, Gemeda MN (2018) Prevalence of Anemia and Associated Risk Factors among Pregnant Women Attending Antenatal Care in Selected Health Centers in Addis Ababa, Ethiopia. J Women's Health Care 7: 443 10.4172/2167-0420.1000443

[28] Soh K.L., Tohit E.R.M., Japar S., Geok S.K., Ab Rahman N.B. and Raman R.A. (2015) Anemia among Antenatal Mother in Urban Malaysia. Journal of Biosciences and Medicines, 3, 6–11. 10.4236/jbm.2015.33002

[29] Acheampong K, Appiah S, Baffour-Awuah D et al Prevalence of anemia among pregnant women attending antenatal clinic of a selected hospital in Accra, Ghana. Int J Health Sci Res. 2018; 8(1):186–193.

[30] Abel G and Afework M. Prevalence of Anemia and Associated Factors among Pregnant Women in North Western Zone of Tigray, Northern Ethiopia, Journal of Nutrition and Metabolism, Volume 2015, Article ID 165430, 7 pages 10.1155/2015/165430. PMC4475559 26137321

[31] Mihiretie H, Fufa M, Mitiku A, Bacha C, Getahun D, et al (2015) Magnitude of Anemia and Associated Factors among Pregnant Women Attending Antenatal Care in Nekemte Health Center, Nekemte, Ethiopia. J Med Microb Diagn 4: 197 10.4172/21610703.1000197

[32] Mohammed Ebrahim, Mannekulih Ephrem, Abdo Mayrema. Magnitude of Anemia and Associated Factors Among Pregnant Women Visiting Public Health Institutions for Antenatal Care Services in Adama Town, Ethiopia. Central African Journal of Public Health. Vol. 4, No. 5, 2018, pp. 149–158. 10.11648/j.cajph.20180405.14

[33] Alene Kefyalew Addis and Dohe Abdulahi Mohamed. Prevalence of Anemia and Associated Factors among Pregnant Women in an Urban Area of Eastern Ethiopia, Hindawi Publishing Corporation Anemia, Volume 2014, Article ID 561567, 7 pages 10.1155/2014/561567. PMC4158560 25215230

[34] Kefiyalew et al: Anemia among pregnant women in Southeast Ethiopia: prevalence, severity and associated risk factors. BMC Research Notes 2014 7:771 10.1186/1756-0500-7-771 25362931 PMC4223834

[35] Bereka SG, Gudeta AN, Reta MA, Ayana LA (2017) Prevalence and Associated Risk Factors of Anemia among Pregnant Women in Rural Part of JigJiga City, Eastern Ethiopia: A Cross Sectional Study. J Preg Child Health 4: 337 10.4172/2376-127X.1000337

[36] Kenea Adamu, Negash Efrem, Bacha Lemi, and Wakgari Negash. Magnitude of Anemia and Associated Factors among Pregnant Women Attending Antenatal Care in Public Hospitals of Ilu Abba Bora Zone, South West Ethiopia, Hindawi Anemia Volume 2018, Article ID 9201383, 7 pages 10.1155/2018/9201383 PMC6257898 30538862

[37] Shridevi. Study of prevalence of anemia among pregnant women attending antenatal checkup in a rural teaching hospital in Telangana, India. Int J Reprod Contracept Obstet Gynecol 2018; 7: 2612–6.

[38] Abriha et al: Prevalence and associated factors of anemia among pregnant women of Mekelle town: a cross sectional study. BMC Research Notes 2014 7:888 10.1186/1756-0500-7-888 25487251 PMC4295569

[39] Zekarias B, Meleko A, Hayder A, Nigatu A, Yetagessu T (2017) Prevalence of Anemia and its Associated Factors among Pregnant Women Attending Antenatal Care (ANC) In Mizan-Tepi University Teaching Hospital, South West Ethiopia. Health Sci J. Vol. 11 No. 5: 529.

