# Supplementary material for: Magnitude and factors associated with anemia among pregnant women attending antenatal care in Bench Maji, Keffa and Sheka zones of public hospitals, Southwest, Ethiopia, 2018: A cross -sectional study
Source: PLoS One. 2019 Nov 21;14(11):e0225148. doi: 10.1371/journal.pone.0225148 (PMC6872185; doi:10.1371/journal.pone.0225148)
Supplement: S1 Table — (DOCX) [file pone.0225148.s001.docx]

**Table S1**. Description of variables and measurement for the study in Bench Maji, Keffa and Sheka zones of public hospitals, Southwest, Ethiopia, 2018.

| **Variables** | **Descriptions** | **Measurements** |
| --- | --- | --- |
| Anemia | Third trimester pregnant mother with hemoglobin level less than 11g/dl. | Pregnant mother with hemoglobin level <11g/dl coded as ‘1’, those with hemoglobin level≥11g/dl coded as ‘0’. |
| Nutritional status | Nutritional status measurement based on Mid-upper arm circumference. If mid-upper arm circumference pregnant woman is ≥21cm considered as normal and if <21cm considered as under nutrition. | Mid-upper arm circumference less than21cm coded as ‘1’ and mid-upper arm circumference ≥21cm coded as ‘2’. |
| Family size | Number of family members | Categorized into three:’≤4’, ‘5-6’ and ‘≥7’. |
| Gravida | Number of pregnancies a woman ever have including  current pregnancy | Categorized in to three: ‘one or primi gravida’, ‘gravid 2-4’  and ‘gravida 5 or above’ |
| Parity | Number of pregnancies reached viability | Categorized in to two: primiparous coded as ‘1’ and multiparous coded as ‘2’. |
| Gestational age | Gestational age was calculated based on the last normal menstrual period (LNMP) and those LNMP unknown, we relied on ultrasonography measures. | Categorized in to two: Gestational age less than 37 weeks coded as ‘1’ and ≥37 weeks coded as ‘2’ |
| Number of ANC visit | Number of having health facility visit for pregnancy check up by skilled attendants during pregnancy.  . | Categorized in to four: ‘one visit’ ‘two visit ‘‘three visit’ and ‘four and above’. |
