# Supplementary material for: Magnitude and factors associated with anemia among pregnant women attending antenatal care in Bench Maji, Keffa and Sheka zones of public hospitals, Southwest, Ethiopia, 2018: A cross -sectional study
Source: PLoS One. 2019 Nov 21;14(11):e0225148. doi: 10.1371/journal.pone.0225148 (PMC6872185; doi:10.1371/journal.pone.0225148)
Supplement: Attachment — Submitted filename: Response to reviewers comments and questions.docx [file pone.0225148.s004.docx]

**Response to Reviewers’ comments and questions**

| **Reviewers’ comment/questions** | **Response to reviewers’’ comments/questions** | remark |
| --- | --- | --- |
| Reviewer #1  - Insufficiency of variables in terms of anemia and its magnitude  - Contemporariness of references | -Of course there are some variables that were not included. But we focused on the variables that accurately measured at health facility during giving services for women at ANC clinic.  -We updated the references for important information |  |
| Reviewer #2   - flow of introduction section - methods section - how outcome coded - result section - What to mean face validity? | -The introduction part was revised  -All hospitals (4 hospitals in the three zones) were included and the study population was corrected as all pregnant women fulfilled the inclusion criteria  -Anemic women coded as 1 and not anemic coded as 0  -Result section revised  -Face validity is one of the validity test by using individuals who have specialty and special training on the area. |  |
| Reviewer #3   - comment 1   -first  -second   - comment 2   -first   - comment 3 - comment 4 - comment 5 - comment 6 - comment 7 - comment 8 - comment 9 - comment 10 - comment 11 - comment 12 - comment 13 - comment 15 - comment 16 - comment 18 - comment 19 - comment 20 - comment 21 - comment 22 - comment 23 - comment 24 - comment 25 - comment 26 - comment 27 - comment 29 - comment 30 - Comment 40 or 31? - comment 41,42 | -the result in abstract was revised  -the introduction part was revised  -corrected  -the sample size proportionally allocated to the 4 hospitals according to their estimated patient flow during data collection time.  -midwives/corrected grammar errors  -as our outcome variable is not multinomial (only anemic and not anemic) - our outcome is binary, so we used binary logistic (multivariable logistic regression).  -under this subtitle we tried to put frequency, because the cross tabulate is to indicate bivariate analysis to show the relationship b/n each indicators and the outcome .this purpose is replaced by crude odds ratio which is in the column of table **5** under the factors associated to anemia.(cross tab is the same to COR in purpose) –cross tabulation of all variables/indicators were presented in **table 5**.  -rephrased and corrected  -we tried to explain on response to comment 7  -corrected  -corrected  -corrected  -corrected  -rephrased  -the same to comment 7&9  -corrected  -revised  -revised  -corrected  Revised  -malnutrition shows both under nutrition and over-nutrition. So, under nutrition (MUAC <=21cm) is correct in place.  -the respondents being anemic were 356 not 514.  -revised  -AOR is to indicate adjusted Odds ratio or multi variables  -COR (crude odds ratio) is bivariate analysis that show the association between each variable and outcome .if the variable associated with outcome by bivariate analysis by p-value < 0.25 that variable is candidate for multi variable analysis  -corrected  -revised  -we used them in literature review section of mother document which used in discussion part. |  |
| Reviewer #4   1. first comment 2. second comment 3. third comment 4. fourth comment 5. fifth comment 6. sixth comment 7. seventh comment 8. eighth comment 9. ninth comment 10. tenth comment 11. eleventh comment 12. twelfth comment 13. thirteenth comment 14. fourteenth comment 15. comment 16. 17. Comment 18. comment 19. comment 20. comment 21. comment 22. comment 23. comment 24. comment 25. comment | -we took the estimation of population for 4 months from each hospitals plan which was 3000 women attending antenatal care. Then based on the above population the sample size proportionally allocated to each hospital.  The pregnant women come to health facility randomly, so that we took only eligible women consecutively while jumping those ineligible one.  -since we took the study subjects consecutively, we jumped the ineligible until sample size achieved, so that the response rate became 100%.  -the 12 midwives (3 for each hospital) worked in shift with the same approach.  -it can be easily understandable from frequency (column 3&4) of table 5.  -the population of the three zones of study area in which the hospitals found have similar in socio economic, geographically and in management style or structure as in the same region ,we didn’t think there is difference in prevalence among the hospitals.  -regarding the educational status classification in this study, the data of read and write (informal education) is not overlapped with educational status. The knowledge of women with only read and write is not the same with primary education.(i.e The data was collected separately).  -the variables (age, residence and gestational age) have no reversed association with anemia after adjustment for confounding (last column of table 5). But before adjustment  -revised  -yes, the cited studies were conducted in hospital setting  Accept the comment -revised  -blood test used to diagnose anemia, the method used as per guide line of our country. The aim of our study is only determine prevalence not to identify the type of anemia.  -after adjustment of confounding teenage age is not associated but older ages groups are associated with higher risk.  -even though gestational age is non-modifiable variables, it gives clue about the importance of starting early iron foliate supplementation and counseling the mother about prevention of anemia in pregnancy  - The common cause of anemia is nutritional deficiency. So family size related with diet (iron content) that the mother may get that may intern affect the biologic (the tendency of hemoglobin in red blood).  The funder this study was Mizan Tepi University  -revised  We used estimated proportion and confidence interval for sample size calculation. We accept the comment but the clients come to hospital randomly.  -Family size and parity have different definition , parity means number of pregnancy reached viability whereas family size means a number of people living together under one roof (in one house). No this much similarity.  -in our context it is obvious that orthodox is Christian, but there are many other religions under Christianity  -family size is not only children it includes mother, father and other relatives who live in the house  -revised  -pregnant mother must get additional diet during pregnancy than before pregnancy .thus to see if mother get diet in addition to previously used diet ,we use that variable.  -if the large size improves the precision, that data explains the population that is generalizability.  -done |  |
| **Reviewer #5** | **Response to comments** |  |
| Naegele's rule' formula to calculate the gestational age. | - We used Ethiopian calendar to calculate the gestational age. - Naegele’s rule is not commonly used in Ethiopia. |  |
| Few grammatical errors need to be corrected. | - All grammatical errors are revised and corrected. |  |
| **Reviewer # 6** |  |  |
| Should be clear on methodology, it is still not clear | - Some part of methodology were revised |  |
| Focus on sample size calculation | - We calculated the sample size by using single population proportion formula and all assumptions were considered. |  |
| Spelling errors | - Spelling errors were revised |  |
