# Supplementary material for: Magnitude and factors associated with anemia among pregnant women attending antenatal care in Bench Maji, Keffa and Sheka zones of public hospitals, Southwest, Ethiopia, 2018: A cross -sectional study
Source: PLoS One. 2019 Nov 21;14(11):e0225148. doi: 10.1371/journal.pone.0225148 (PMC6872185; doi:10.1371/journal.pone.0225148)
Supplement: Attachment — Submitted filename: Reviewer comment on anemia to plos one.docx [file pone.0225148.s003.docx]

| S No | Section | Gaps | Suggestions |
| --- | --- | --- | --- |
|  |  | Medical related illness, pregnancy status (intended and unintended, Pregnancy related Complications, physical harassment |  |
| 1 |  | Address of corresponding author  Mizan-Tepi University  Addis Abeba/Finfine, ETHIOPIA | Mezan Tepi is not part of Addis Ababa  (put the address correctly) |
| 2 | Title | Seems incomplete | **Magnitude and factors associated with anemia among pregnant women** attending ANC in public  Hospitals of Benchi-Maji, Kaffa and Sheka zones, Southwest, Ethiopia |
| 3 | Abstract | It also brings different obstetrical problems like; prematurity, low birth weight,  abortion, intrauterine fetal death and perinatal mortality and other maternal health problems like;  impaired immune function, poor work capacity, fatigue, increased risk of cardiac diseases and  mortality. | Redundant, you already mention this idea “maternal and perinatal diverse consequences and it increase the risk of maternal and perinatal  mortality. “ |
|  |  | All third trimester pregnant women attending antenatal care at Mizan-Tepi University Teaching Hospital, Tepi, Gebretsadik Shawo and Wacha public hospitals were included in the study. | No time bound stated, and your sample size was 1871, so I hope the total third trimester mothers were not included so revise it |
|  |  | educational status [AOR 2.06, 95% CI (1.12-3.80)], gestational age [AOR 1.94, 95% CI (1.27-2.96)], nutritional status [AOR 3.00, 95% CI (2.22- 3.97)] and family size [AOR 2.66, 95% CI (1.49-4.77)] | Direction of association was not clearly stated for e.g. the educated or not educated were at risk of anemia???, HH with low or high family size??? and so on…  Show us direction  same as in the conclusion section |
| 4 | Introduction |  | Needs to have some sort of flow (definition of anemia, how it is been assessed, the magnitude and then the gap (why you were interested to study) |
| 5 | Methods |  |  |
|  | Study area & setting | Not only name of hospitals | At least you are expected to show us the number of health facilities, coverage on |
| 6 | Study population: | all sampled pregnant women ae not the study population. | Sampled populations are populations taken from your study population (i.e. sample size) so define your study population |
|  | Sample size determination | p**=**proportion expected prevalence of anaemia are 23% (Limo district Ethiopia) | Put the source (reference) not limo district Ethiopia |
| 7 | Sampling technique | ***How you selected not stated clearly***  the average was considered as  source of population.  The study participants were consecutively taken from each hospital till the  sample size was achieved. | Were the 4 hospitals selected randomly? If yes you are expected how did you selected them from how many hospitals or health facilities? You also need to state when and how you selected participants |
| 8 | **Data collectors** | Twelve data collectors who were degree midwife were recruited | Rewrite it could be Bachelor degree holder midwives |
| 9 | Data collection procedure | Gestational age was calculated based on the last normal menstrual period (LNMP). When LNMP-based gestational age is unknown, we relied on ultrasonography measures. | Which one was best to confirm GA (LNMP or ultrasonography) please describe how you proceed?  Please describe clearly the SOP how blood sample was drawn, transported, stored and so on |
| 10 | **Data Quality control measures** | pre-test was done on 5% of the total study eligible subjects | Did you pretest using your study participants and then exclude them or did you mean pretest was done on 5% of sample size from non-sampled mothers?? |
|  |  | The validity of the tool was checked by face validity. | What to mean face validity?? |
| 11 | **Result** | Out of the total 1871 expected participants, 1871 participants participated in the study which  brings response rate of 100%.   - Around half 853(45.6%) - majority 1483(79.3%) - Most1808(96.7%) | Rewrite it precisely and don’t put the words like study participants (obvious they are your study participants)  You could write it as  All the sampled mothers were participated (100% response rate)  –choose either e.g. majority (79.3%) or 1483 (79.3% |
| 12 | **Table 5** | Regression analysis  What you stated in the text and what is in the table were different  Most of the OR values computed were not compatible based on the OR formula of AD/BC  There could be a very rare possibility for a variable which was protective in the bivariable regression become a risk factor in the multivariable regression model  For example, age of respondent & gestational age. But this is mainly due to problem of referencing (not being consistent in using the same reference, either first or last) in the two models | Please recalculate COR considering your outcome of interest!!!  Your focus is to identify the factors why people get anemic during pregnancy!!  If this is the case then your A is the values in the Yes column and your B is the No column.  Therefore, please reanalyze your regression.  For example, based on your Table, pregnant mothers who read and write were 2 times more likely to be non-anemic compared to those mothers who attend diploma & above. **2.06 (1.12-3.80) ***  e.g. 2, mothers who took alcohol occupationally were 2 times more likely to be non-anemic than those who never drink alcohol (**2.01(1.14-3.55)** |
